# Meetings of Societies

**Published:** 1950-10

**Authors:** 


### Meetings of Societies

### Bristol Medico-Chirurgical Society

Programme, 1950–51

October. Annual General Meeting: President's Address.

November. Clinical Meeting.

December. "**Geriatrics.**" Dr. C. T. Andrews, Dr. T. S. Wilson, and Dr. W. Hughes.

January. "**The Medical Aspects of Atomic Bombing.**"

February. "**Intestinal Obstruction,**" Mr. Dickson Wright.

March. Two short papers—"**Some Rare Diseases Contracted from Animals,**" Dr. A. M. G. Campbell; and "**Eczema of the Colon,**" Dr. J. Naish.

April. (*To be announced later*).

May. "**Surgical Trends in America,**" Mr. Howard K. Gray, Mayo Clinic.

### The Clinical Society of Bath

*Officers*, 1950–51: President, Mr. R. Colley; President Elect, Dr. D. G. Steven; Hon. Treasurer, Dr. E. Scott-White; Hon. Secretary, Mr. A. Daunt Bateman.

The first meeting will take place at the Royal United Hospital at 8.30 p.m. on Friday, October 13th, when Dr. C. Hagenbach will read a paper entiled "**Allergies in General Practice**". Meetings will be held on the second Friday in each month.

### Cardiff Medical Society

1950–51

*Officers:* President, T. Wallace; Vice-President, H. L. Coulthard; Treasurer, R. G. Maliphant; Editor, J. D. Spillane; Secretaries, E. Williams, 74 Penylan Road, B. Evans, 59 Cathedral Road.

October 10th. Presidential Address: "**Twenty Years' Experience as a Prison Medical Officer**".

Meetings on second Tuesday of each month.

### Devon and Exeter Medico-Chirurgical Society

*Officers:* President, Dr. Charles Wroth; Vice-President, Dr. L. N. Jackson; Secretary, Dr. Charles Seward; Reporting Secretary, Dr. L. N. Jackson; Treasurer, Dr. C. P. Warren.

*Council:* Drs. J. T. Dunkerley, John Heel, D. T. Mackie, P. A. Hederman, H. A. Constable, M. Dykes Bower, W. H. Jones.

Meetings are held on every third Thursday and some first Thursdays, October–April in the afternoon or evening. The meetings are clinico-pathological demonstrations, clinical meetings and lectures.

### Torquay and District Medical Society

1950–51

*Officers:* President, Mr. W. Etherington-Wilson; Hon. Secretary, Dr. R. A. Lattey, Ambrook, Rousdown Road, Torquay (Tel.: Torquay 65243); Hon. Treasurer, Dr. J. R. Imrie.

Unless otherwise stated, meetings are at the Torbay Hospital at 8.30 p.m.

October 26th. 4.30 p.m. Opening Meeting. Sir James Paterson Ross, K.C.V.O., "**Tuberculous Lymphadenitis**". 7.45 p.m. Dinner and Dance, Palace Hotel, Torquay.

November 9th. Clinical Discussion.

November 23rd. Dr. E. C. Warner, F.R.C.P., "**Medical Aspects of Hypertension**".

December 14th. Norman Lake, Esq., F.R.C.S., "**Surgical Aspects of Hypertension**".

January 11th, 1951. 4.30 p.m. Clinical Meeting.

January 25th. Kenneth Pridie, Esq., F.R.C.S., "**Backache, Sciatica and Ruptured Discs**".

February 8th. Clinical Discussion.

February 22nd. Dr. Denis Williams, F.R.C.P., "**Faints and Fits**".

March 8th. Dr. E. R. Cullinan, "**Chronic Diarrhoea**".

March 21st. Dr. C. J. C. Britton, "**Allergy from the Practical Point of View**".

April 12th. 4.30 p.m. Newton Abbot Hospital. Clinical Meeting.

April 26th. Alan Brews, Esq., F.R.C.O.G., "**The Changing Face of Midwifery**".

In June, Summer Meeting at Hawk Moor Sanatorium.

### Cornwall Clinical Society

*Officers:* President, G. P. O'Donnell, M.B., B.Ch.; Joint Honorary Secretaries, L. W. Hale, M.D., F.R.C.P., F. D. M. Hocking, M.Sc., M.B., B.S.

The society meets alternately at the Royal Cornwall Infirmary, Truro, and at Redruth Hospital.

### Wessex Rahere Club

*Officers:* President, Sir Holburt Waring; Chairman, Professor R. J. Brocklehurst; Hon. Secretary, Mr. A. Daunt Bateman.

It is hoped that the Autumn Dinner will be held at the Spa Hotel, Bristol, on Saturday, October 21st. Any old Bart's men who are not yet members are invited to get in touch with the secretary.

### British Medical Association: Gloucestershire Branch

*Officers:* President, Mr. J. F. H. Stallman, F.R.C.S.; Secretary, Dr. E. W. Hyde.

### Bristol Division

*Officers* (1950–51)*:* Chairman, Dr. G. E. F. Sutton; Chairman Elect, Dr. T. Milling; Secretaries, Dr. H. M. Golding, 10 Barton Hill Road, Bristol 5, Dr. W. H. Hayes, 9 Logan Road, Bishopston, Bristol 7; Treasurer, Dr. C. Dix.

*Committee:* Drs. H. K. V. Soltau, D. M. Cameron, J. M. Evans, Jnr., D. E. Olliff, A. M. MacLachlan.

